# Delusional parasitosis with hyperthyroidism in an elderly woman: a case report

**DOI:** 10.1186/1752-1947-7-17

**Published:** 2013-01-10

**Authors:** Eylem Ozten, Ali Evren Tufan, Cem Cerit, Gökben Hızlı Sayar, Irem Yalug Ulubil

**Affiliations:** 1 Department of Psychiatry, Uskudar University Medical Faculty, Istanbul, Turkey; 2 NPİstanbul Nöropsikiyatri Hastanesi, Alemdağ Cad., Site Yolu, No: 29, 34768, Umraniye, Istanbul, Turkey; 3 Department of Child and Adolescent Psychiatry, Abant Izzet Baysal University, Medical Faculty, Bolu, Turkey; 4 Department of Psychiatry, Kocaeli University Medical Faculty, Izmit, Turkey

## Abstract

**Introduction:**

Delusional parasitosis is a rare, monosymptomatic psychosis involving a delusion of being infested with parasites. It is commonly observed among female patients over the age of 50. It is classified as a ‘delusional disorder’ according to the 10^th^ revision of the International Classification of Diseases and as a ‘delusional disorder - somatic type’ according to the Diagnostic and Statistical Manual, Fourth Edition. Delusional parasitosis was reported to be associated with physical disorders such as hypoparathyroidism, Huntington’s chorea and Alzheimer’s disease, among others. Other than vitamin deficiencies however, a causal relationship has not to date been identified. We present this case due to the rarity of Turkish patients with this condition, its duration of follow-up, and its temporal pattern of symptoms paralleling thyroid function tests.

**Case presentation:**

Our patient was a 70-year-old white Anatolian Turkish woman with primary school education who had been living alone for the past five years. She presented to our psychiatry department complaining of ‘feeling large worms moving in her body’. The complaints started after she was diagnosed with hyperthyroidism, increased when she did not use her thyroid medications and remitted when she was compliant with treatment. She was treated with pimozide 2mg/day for 20 months and followed-up without any antipsychotic treatment for an additional nine months. At her last examination, she was euthyroid, not receiving antipsychotics and was not having any delusions.

**Conclusion:**

Although endocrine disorders, including hyperthyroidism, are listed among the etiological factors contributing to secondary delusional parasitosis, as far as we are aware this is the first case demonstrating a temporal pattern of thyroid hyperfunction and delusions through a protracted period of follow-up. It may be that the treatment of delusional parasitosis depends on clarifying the etiology and that atypical antipsychotics may help in the management of primary delusional parasitosis. Further studies on the relationship between thyroid hormones and dopaminergic neurotransmission may be warranted.

## Introduction

Delusional parasitosis (DP) is a monosymptomatic psychosis involving a delusion of being infested with parasites [1]. Patients with DP believe that insects, worms or other small pests live in their bodies and feed on them [2]. Visual or tactile hallucinations consistent with thought content can accompany the delusions. DP is typically observed in women over the age of 50 although isolated cases among men have been reported [1-3]. It can be classified as primary or secondary according to etiology. Primary DP is a psychiatric disorder whereas secondary DP is currently thought of as a symptom rather than a disorder. In the latter case, the delusion is secondary to another psychopathology or a medical illness [4,5].

Secondary DP has been associated with diseases and malignancies of the hematopoietic, pulmonary, cardiac, renal, gastrointestinal and endocrine systems, infections of the central nervous system, and nutritional deficiencies including vitamin B12, folate and pellagra [1-5]. However, the causal role of the underlying disorder has rarely been elucidated [1-5].

Although thought to be rare, the true prevalence of DP may be underestimated because patients do not believe the psychiatric origin of their complaints and are reluctant to apply for psychiatric treatment. Most cases are reported from Western countries with recent reports from Asia. Although the number of reported cases increases steadily, it remains a rare phenomenon. Here, we report the treatment of a patent with DP thought to be secondary to hyperthyroidism. This case was deemed worthy of presentation due to the scarcity of cases from Turkey, its duration of follow-up, and its temporal pattern of symptoms paralleling thyroid function tests.

## Case presentation

Our patient was a 70-year-old white Anatolian Turkish woman with primary school education who had been living alone for the past five years. She presented to our Psychiatry Department complaining of ‘feeling large worms moving in her body’. The complaint had been present for the past six months and started after she presented to an internist with complaints of weight loss, bilateral fine tremor of the upper extremities, shaking, sweating, palpitations and intolerance to heat. After the evaluation, she was hospitalized for a diagnostic work-up in our Endocrinology Department with a preliminary diagnosis of hyperthyroidism. Restlessness, increased speech and the feeling of ‘large worms migrating through her body’ were observed on the second day of hospitalization.

After psychiatric consultation, our patient was diagnosed with a psychotic disorder due to a general medical condition (hyperthyroidism). She was started on 2mg/day of haloperidol. This treatment was chosen because of the preliminary nature of the diagnosis and because our patient may have had delirium. Thyroid ultrasonography revealed five nodules measuring 2mm to 5mm in the left lobe and a heterogeneous nodule measuring 5mm to 10mm in the right lobe. None of the nodules displayed microcalcification, solidity or a reduced echogenicity pattern. The solitary nodule in the right lobe displayed an irregular contour. Lymphadenopathy and invasion of the neighboring structures were not noted. Scintigraphy revealed that all of the nodules were hyperfunctioning. She had no history of exposure to ionizing or environmental toxins, and no personal or family history of multiple endocrine neoplasia. A fine needle aspiration biopsy ruled out malignancy. The level of antibody to thyroid stimulating hormone receptor was found to be 2.5IU/L, thereby confirming Graves' disease.

As a result of the surgical consultation, medical follow-up was advised. On the third day, propylthiouracil 150mg/day and propranolol 30mg/day were started and the doses were maintained at 100mg/day and 40mg/day, respectively, after the first week. Restlessness, increased speech and DP remitted on the 10th day of treatment and haloperidol was stopped. She was discharged on the 20th day with a prescription of levothyroxine sodium 100μg/day (weight, 59kg; dose, 1.7μg/kg/day). A fortnight after discharge, she started to feel the worms. After presenting to the Departments of Dermatology, Microbiology and Infectious Diseases, Endocrinology, and Neurology, she was referred to the Department of Psychiatry and hospitalized. Her past medical and psychiatric histories were negative for pathology and her family history did not reveal evidence of psychopathology. She was teetotal and she was receiving no drugs or supplements at the time of evaluation other than levothyroxine sodium. She had no history of drug use or abuse and an inpatient follow-up lasting a month did not reveal any signs or symptoms of withdrawal.

At the baseline examination, she was noted to be cooperative and have reduced self-grooming. Her speech was fluent although restricted to the topic of tapeworms. Her mood and affect were anxious. She was orientated to people, space and time. Her attention, concentration and memory were within normal limits. She reported tactile hallucinations. Her judgment, reality testing and abstract thinking were impaired and her thought process was perseverative and circumferential. Her thought content was positive for somatic delusions. Motor retardation was observed while sleep and appetite were normal. Assessment with the Positive and Negative Syndrome Scale and Mini Mental State Examination revealed scores of 82 (positive: 19; negative: 18; general psychopathology: 45) and 28, respectively.

Laboratory examinations including levels of vitamin B12, folate and ferritin, the Venereal Disease Research Laboratory test, and a toxicology screen were within normal limits other than elevated thyroid function tests (our patient was noncompliant with her levothyroxine treatment, Table 1). After an endocrinology consultation, it was advised that she be followed without medication for a month; thereafter, levothyroxine sodium 100μg/day was restarted. Consultations from the departments of Internal Medicine, Neurology, Hematology and Oncology ruled out the presence of an organic disorder. Electroencephalography and cranial magnetic resonance imaging were normal. Therefore our patient was diagnosed as having DP, presumably secondary to the hyperthyroidism. A baseline electrocardiogram (ECG) demonstrated a corrected QT interval (QTc) duration of 450ms. Previous ECGs revealed that her QTc duration varied between 440ms and 490ms. Therefore, psychiatric treatment was initiated with pimozide 2mg/day and later the dose was titrated to 4mg/day. At the eighth week of treatment her thyroid function tests returned to normal limits along with a remission of the delusions. The QTc duration at this time was measured as 470ms. Our patient was discharged from the hospital and followed-up at the outpatient department. Her thyroid levels remained normal for a year and she was free of psychiatric symptoms. Therefore, pimozide was titrated down to 2mg/day. After 15 months her thyroid hormone levels increased again due to an irregular use of medication. Consequently, she began to feel the tapeworms again.

**Table 1:** The relationship of thyroid function tests with positive and negative syndrome scale scores and psychiatric symptoms in a patient with delusional parasitosis

**Date**	**Triiodothyronine**^**a**^**(ng/dL)**	**Free thyroxine**^**b**^**(ng/dL)**	**Thyroid stimulating hormone**^**c**^**(mIU/L)**	**Delusions**	**PANSS (positive/negative/general)**
Baseline^d^	6.55	2.27	0.007	+	54 (14/9/31)
Month 6	7.95	3.97	0.005	+	82 (19/18/45)
Month 9	3.78	2.19	1.28	-	44 (7/12/25)
Month 15	3.56	1.97	1.32	-	38 (7/8/23)
Month 25	4.58	2.47	0.09	+	68 (15/16/37)
Month 29	3.97	2.07	1.02	**-**	34 (7/7/20)

^a^ Normal range: 0.2 to 0.5ng/dL; ^b^ normal range: 0.7 to 1.8ng/dL; ^c^ normal range: 0.3 to 4.5mIU/L; ^d^ baseline without levothyroxine - all other levels with 100μg/day levothyroxine. PANSS: Positive and Negative Syndrome Scale.

Her complaints disappeared when her anti-thyroid medication was reinstituted. After 20 months of follow-up the pimozide was stopped and she was followed without antipsychotic treatment for an additional nine months. At her last examination she was euthyroid, not receiving antipsychotics and having no delusions.

## Discussion

We have described a case of DP thought to be due to hyperthyroidism and reported its treatment and follow-up. Many individuals with DP are functional, with a minority being severely affected. The distress and loss of functionality reported in our patient shows that she was severely affected [1]. It has been reported that patients commonly present for general medical or dermatologic care, onset is insidious and most patients report symptoms lasting at least six months, the diagnosis may take years to establish, the belief of infestation may be shared, and that patients do not generally have a history of prior psychopathology but a history of social isolation [6,7]. These features were also present in our patient.

Hypochondriasis, dementia, mood and anxiety disorders, obsessive compulsive disorder and post-traumatic stress disorder were ruled out because of the intensity of conviction, her normal neurological and laboratory evaluations, the lack of previous history for psychiatric disorders and the absence of traumatic stressors in our patient.

Pimozide was used to treat our patient because, until recently, it was recommended for use in DP. However, it is currently not used as a first-line antipsychotic for patients with DP because of the risk of extrapyramidal symptoms, longer QTc interval and interactions with other drugs. Although our patient did not experience untoward side effects due to interactions with other agents or extrapyramidal side effects, follow-up ECGs revealed longer QTc intervals, illustrating the potential for cardiotoxicity. Several case reports have indicated the beneficial effects of atypical antipsychotics in primary DP, such as risperidone, quetiapine, olanzapine and amisulpride [6-10].

The use of haloperidol at 2mg/day at the initial presentation may be an important limitation of our case report. Haloperidol at this dose may not affect delusions and may lead to increased agitation. However, it was chosen at this time pending the results of laboratory tests and to rule out the presence of delirium. The current consensus is that pharmacological treatment of delirium is necessary if the patients pose a safety risk to themselves or others and that both typical and atypical antipsychotics are effective in the management of delirium, although they lack approval from the US Food and Drug Administration for this indication [11].

The pathogenesis of DP is currently unknown; proposed factors include recent awareness of a known disease via media or another individual, leading to amplification of somatic sensations; an elevation of extracellular dopamine within the striatum; social isolation; or stress [1-5,7]. Social isolation and stress of the diagnosis of hyperthyroidism may have played a role in our patient but the temporal relationship between thyroid hormone levels and the delusion of parasitosis (Table 1) may also point to a role of thyroid dysfunction in the development of delusion.

## Conclusion

Although endocrine disorders, including hyperthyroidism, are listed among the etiological factors contributing to secondary DP, as far as we are aware this is the first case presentation demonstrating a temporal pattern of thyroid hyperfunction and delusions through a protracted period of follow-up. It may be that treatment of DP depends on clarifying the etiology and that atypical antipsychotics may help in the management of primary DP. Further studies on the relationship between thyroid hormones and dopaminergic neurotransmission may be warranted.

## Consent

Written informed consent was obtained from the patient for publication of this case report and accompanying images. A copy of the written consent is available for review by the Editor-in-Chief of this journal.

## Abbreviations

DP: Delusional parasitosis; ECG: electrocardiography; QTc: corrected QT interval.

## Competing interests

The authors declare that they have no competing interests.

## Authors’ contributions

EO, CC, AET, GHS and IYU diagnosed and followed the patient. AET and EO prepared the manuscript and CC, GHS and IYU evaluated the draft and suggested revisions. All authors read and approved the final manuscript.
